# Pathogenicity & virulence of *Histoplasma capsulatum* - A multifaceted organism adapted to intracellular environments

**DOI:** 10.1080/21505594.2022.2137987

**Published:** 2022-10-26

**Authors:** Alessandro F. Valdez, Daniel Zamith Miranda, Allan Jefferson Guimarães, Leonardo Nimrichter, Joshua D. Nosanchuk

**Affiliations:** a Departamento de Microbiologia Geral, Universidade Federal do Rio de Janeiro, Instituto de Microbiologia Paulo de Góes, Rio de Janeiro, Brazil; b Departments of Medicine (Division of Infectious Diseases) and Microbiology and Immunology, Albert Einstein College of Medicine, Bronx, NY, USA; c Departamento de Microbiologia e Parasitologia - MIP, Universidade Federal Fluminense, Instituto Biomédico, Rio de Janeiro, Brazil

**Keywords:** *Histoplasma capsulatum*, dimorphism, virulence, intracellular parasitism, histoplasmosis

## Abstract

Histoplasmosis is a systemic mycosis caused by the thermally dimorphic fungus *Histoplasma capsulatum*. Although healthy individuals can develop histoplasmosis, the disease is particularly life-threatening in immunocompromised patients, with a wide range of clinical manifestations depending on the inoculum and virulence of the infecting strain. In this review, we discuss the established virulence factors and pathogenesis traits that make *H. capsulatum* highly adapted to a wide variety of hosts, including mammals. Understanding and integrating these mechanisms is a key step toward devising new preventative and therapeutic interventions.

## Introduction

*Histoplasma capsulatum* is a thermally dimorphic ascomycete with a worldwide distribution. The disease caused by this pathogen is called histoplasmosis, and can affect both immunocompetent and immunocompromised individuals [1,2], hence its definition as a primary pathogen. *H. capsulatum* is found as a mycelium microscopically forming micro and macroconidia in the environment and usually found in soil enriched by nitrogen, commonly preventient from bird and bat droppings [1]. Disturbance in contaminated soil causes mycelia fragments and microconidia to disperse in the air. Upon inhalation by the host, these particles can adhere to the respiratory tract or reach the lungs and regardless of the location, the temperature shift will trigger the filamentous-yeast morphological transformation [2,3].

Distinct factors such as the immune status, the size of the inoculum and virulence of the infecting strain play a role in the prognosis of histoplasmosis [2–30]. In immunocompetent individuals, histoplasmosis is usually benign and asymptomatic (>95%) but eventually can evolve to acute pulmonary disease, with flu-like symptoms within 3 weeks from exposure, such as fever, chills, dry cough, and headaches [31–33]. Although rare in immunocompetent, disseminated histoplasmosis is commonly associated with immunocompromised patients, particularly those displaying T CD4+ deficiency [34]. The symptoms include fever, weight loss, and respiratory manifestations, but overall, are not specific and multiple organs can be affected [35]. During disseminated histoplasmosis maculopapular rash and oropharyngeal ulcers are found in 50% of the cases [36]. In addition, histoplasmosis is frequently misdiagnosed, which may lead to chronic or latent disease [2–37].

There are some basic criteria that pathogenic organisms should meet in order to promote disease, especially in immunocompetent individuals, including: (a) thermotolerance, the ability to grow at or above host temperature, (b) the capacity to invade or bypass host barriers reaching internal tissues, (c) their capacity to evade or withstand the immune system, and (d) their diverse enzymatic repertoire used to acquire nutrients by hydrolyzing and consuming components of the human tissue [38]. In this review, we will discuss the mechanisms by which *H. capsulatum* satisfies these four requirements and describe its well-established virulence factors, including some of its novel ones that, taken together, enable pathogenesis.

## Phylogeny and epidemiology

Since its discovery in 1906 by Dr. Samuel Taylor Darling [39], and its recognition as a fungus in 1912 [40], *H. capsulatum* has historically been divided into three groups according to different geographic distributions, clinical manifestations, and morphologies [41] (i) *H. capsulatum* var. c*apsulatum*, the most common across the world, responsible for classic histoplasmosis, (ii) *H. capsulatum* var. *duboisii*, also a human pathogen, restricted to Central and West Africa and (iii) *H. capsulatum* var. *farciminosum*, an animal pathogen, is responsible for equine histoplasmosis [41]. The literature also classifies *H capsulatum* isolates into two distinct chemotypes (I and II), according to their ability to produce α-1,3-glucan [42]. More recently, Kasuga *et al*. performed a phylogenetic analysis of 137 individual isolates from 25 different countries in 6 continents, representing the three original *Histoplasma* classifications [43], leading to the recognition of 8 clades within 7 phylogenetic species: (i) North American class 1 clade; (ii) North American class 2 clade; (iii) Latin American group A clade (LAm A); (iv) Latin American group B clade (LAm B); (v) Australian clade; (vi) Netherlands clade; (vii) Eurasian clade and (viii) African clade [41,43]. Through an increased *Histoplasma* sampling, Teixeira *et al*. have additionally proposed several subclades of LAm A and LAm B: LAm A1, LAm A2, RJ, and LAm B1 and LAm B2, respectively [44].

In North America, histoplasmosis is endemic in the United States, with most cases being associated with the Ohio and Mississippi River Valleys [35]. It is also the most prevalent systemic mycosis in Central America countries [45] and largely disseminated throughout South America, where it is, likewise, endemic, with several cases reported in Brazil, Ecuador, Venezuela, Paraguay, Uruguay, and Argentina [2,35,45]. Due to the frequent association with HIV patients, in some parts of the globe the histoplasmosis has been considered an AIDS-defining illnesses [46]. Some reports suggest that HIV-associated histoplasmosis has a higher mortality rate than HIV-associated tuberculosis in Latin America [47]. Furthermore, in parts of Latin America, histoplasmosis is often misdiagnosed as tuberculosis, which may inflate tuberculosis statistics [34,48], and mask the real burden of histoplasmosis. In Asia, histoplasmosis is endemic in China, with most cases occurring along the Yangtze River [49], and there are also a high number of cases reported in Thailand, South Korea, and India [30,50,51]. Epidemiological data is scarce in Africa, but there are reports of histoplasmosis in countries, such as Uganda, South Africa, and Tanzania [52–54]; however, this lack of data is mostly because diagnosis still primarily relies on classical mycological diagnosis with fungal culture and microscope techniques, as antigen and PCR tests are not available in most of these countries [55]. Remarkably, there are only 470 cases reported in the last six decades, and there is a growing concern that the disease is overlapping with tuberculosis and HIV in Africa [37,55]. Histoplasmosis is rarely reported in Europe, and most cases are associated with travels to endemic areas [30–58].

### COVID-19 associated histoplasmosis: An important note

Most of the deaths caused by SARS-CoV-2 occur due to lung damage [59]. However, secondary pulmonary or non-pulmonary infections have been reported as major players, including invasive fungal infections (IFI) [59,60]. The main fungal diseases associated to COVID-19 include candidiasis, aspergillosis, murcomycosis, pneumocystosis, cryptococcosis, and histoplasmosis [59]. To date, nine reported cases of histoplasmosis have been associated with Covid-19, three cases in Brazil, two in the United States, two in Argentina, and one in India [61–69].

The number of histoplasmosis-Covid-19 associated cases seems to be low, especially compared to the high-reported numbers of aspergillosis, murcomycosis and candidiasis [59]. Nevertheless, the clinical nature of histoplasmosis and the diagnostic technique limitations are restrictive factors that could lead to low sensitivity/specificity and misdiagnosis [65]. Thus, it is possible that the low number of reported cases of histoplasmosis associated with covid-19, especially in highly endemic areas of North America, is most likely the result of the chaos generated by the pandemic and that the available numbers are probably underreported. It is important to highlight that physicians should be aware of the immunosuppressive nature of SARS-CoV-2 and consider an IFI diagnosis for patients with suggestive signs and symptoms, especially those who experience unexplained clinical worsening in COVID-19 [59].

## Diagnosis and treatment

Diagnosis of histoplasmosis requires a multifaceted approach, including laboratory and epidemiological evidences [70]. The gold standard is the isolation of *H. capsulatum* in culture and observation using direct microscopy from body fluids or tissue specimens. In addition to morphological cues, fluorescent stains, such as Calcofluor, which binds to chitin, the major component of most fungal cell walls, are particularly useful, though non-specific, to aid in the identification of *H. capsulatum* in clinical specimens [70], as opposed to Gram stain, which works poorly for *H. capsulatum*, being more useful for extracellular pathogens, such as *Cryptococcus a*nd *Candida* [70]. Besides, *H. capsulatum* growth can take several weeks, which is especially critical from the clinical point of view. In addition, obtaining adequate tissue or fluid samples for histopathology can be difficult [70]. Antigen detection is a rapid and non-invasive method that provides results with high sensitivity thus not only accelerating the therapeutic intervention but also being a useful marker for clinical follow-up and treatment response [70]. Interestingly, while molecular testing has turned out to be the gold standard for the diagnosis of many pathologies, it has not yet been the case for *H. capsulatum*, as many of the proposed methods perform poorly in comparison to other available methods to this date, resulting in no FDA-approved PCR-based tests for histoplasmosis [70,71].

The clinical management of the patient varies according to the status of the immune system and the progression of the disease. Essentially, antifungal treatment is not indicated for mild cases unless the patient is immunocompromised or symptoms persist for more than four weeks [71]. In moderate cases, the recommended treatment is itraconazole, with voriconazole and posaconazole as additional options. For severe presentations of histoplasmosis, the administration of liposomal amphotericin B is indicated as the primary treatment agent [71–73].

## Pathogenesis

**Figure 1.**
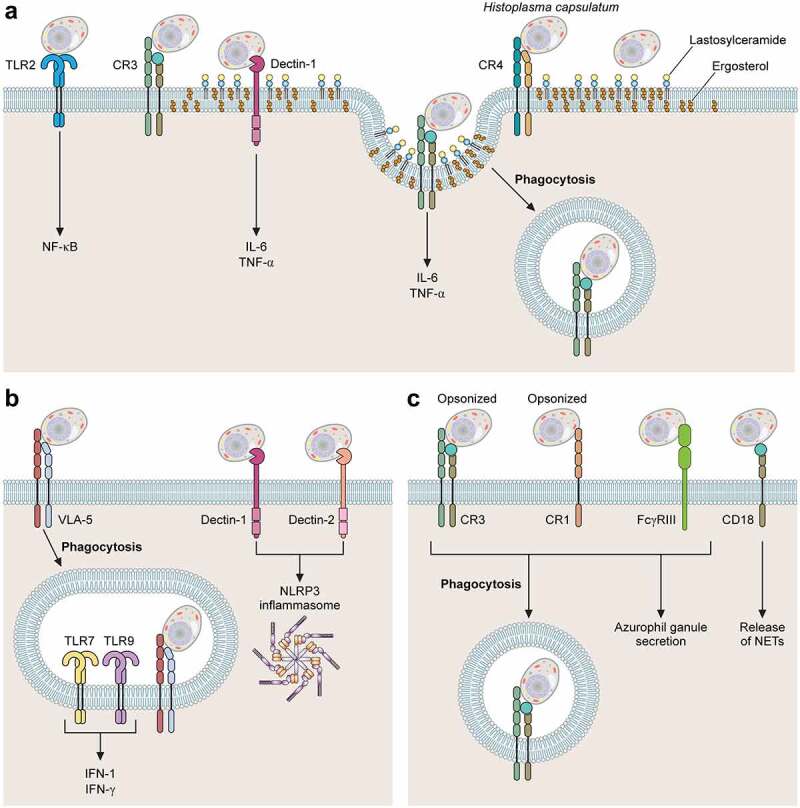
Major receptors involved with H. capsulatum recognition by phagocytes. Association of H. capsulatum with phagocytes. (A) TLR2, CR3, CR4, Dectin-1 and Lactosylceramide (LacCer) participate in the recognition of yeasts of H. capsulatum by macrophages. TLR2 and Dectin-1 are involved with activation of NF-kB and production of cytokines IL-6 and TNF-α, respectively. During H. capsulatum adhesion and internalization, CR3 is recruited to lipid microdomains. (b) Dendritic Cells interact with H. capsulatum using plasma membrane receptors VLA-5, CR3, Dectin-1 and Dectin-2. VLA-5 is the main receptor involved in phagocytosis of H. capsulatum yeasts, CR3 is involved with fungal phagocytosis and Dectin 1 and 2 with the activation of NLRP3 inflammasome. Intracellular receptors TLR7 and TLR9 are involved with production of IFN-1. (c) in neutrophils, the major receptors for ic3b-opsonized H. capsulatum are CR1 and CR3 while non-opsonized yeasts are recognized by FcγRIII, NET release is dependent on CD18. the fungal structures involved with each host cell receptor are shown in Table 1.

The onset of infection occurs through the inhalation of microconidia or mycelial fragments by the host. This step is followed by the deposition of fungal propagules into the pulmonary alveoli and a rapid conversion to the yeast morphology (Figure 1), triggered by the fungal response to the temperature switch. *H. capsulatum* yeasts have an optimal growth at 37 ºC. Morphological changes can occur even before microconidia reach the lungs or within the intracellular environment [1,74]. Pathogenesis results from the initial adaptation to the host environment followed by a successful interaction with the innate immune system. *H. capsulatum* can survive the intracellular environment of phagocytes and evade the effector responses of the host immune system, multiplying, and being carried to the lymph nodes, where they gain access to blood circulation, and can spread to different tissues and organs, characterizing histoplasmosis in its invasive form [2]. *H. capsulatum* combines virulence factors and regulators (Table 1) that are effectively used to defeat the host’s innate and adaptive immune response (Figure 1). The main mechanisms used by this pathogen to survive within the host will be discussed in this section.

**Table 1.** Histoplasma capsulatum virulence factors and regulators.

Surviving in host temperature			Ref
Cysteine and sulphhydryl compounds	Reactivation of mitochondrial respiration by “shunt pathways”		(Maresca [4], Sacco [5])
*DRK1*	Directly associated with hypha-to-yeast transition process, responsible for yeast growth and expression of other proteins		(Nemecek [6])
*Ryp 1, Ryp 2, Ryp 3* and *Ryp 4*	Key regulators of the yeast-phase transcriptional program		(Inglis [7]; Beyhan [8]; Edwards [9])
Bypassing host barriers			Ref
Surfactant proteins of the Host (Collectins SP-A and SP-D)	Direct antifungal activity	*H. capsulatum* evades collectins by invading macrophages	(McCormack [10], Carreto-Binaghi [11])
Evading the immune system			Ref
Hsp60	Mediates yeast recognition and internalization by CR3 receptor of macrophages and has potent immunogenic activity		(Gomez [12], Long [13], Guimarães [14], Guimarães [15], Guimarães [16])
α-1,3-glucan	Impairs recognition of β-1,3-glucan by Dectin-1		(Rappleye [17], Garfoot [18], Ray and Rappleye [19])
Eng1	Acts associated with α-1,3-glucan, hydrolysing any exposed β-1,3-glucans in the cell wall, avoiding recognition by Dectin-1		(Garfoot [18,20])
Sod3, CatB and CatP	Promotes ROS resistance		(Holbrook [21], Youseff [22], Holbrook [23])
*CBP1*	Required for induction of integrated stress response, leading to apoptosis of macrophages		(Isaac [24], English [25], Azimova [26])
Cyclophylin A	Recognized by VLA-5 in DCs leading to yeast internalization		(Gomez [27])
Nutrient Acquisition			Ref
*PCK1*	Encodes a phosphoenolpyruvate carboxykinase, related to the first step of gluconeogenesis,essential to intracellular growth and virulence		(Shen [28])
Ctr3	Copper transporter required for intracellular proliferation		(Shen [29])

### Surviving in host temperature: Key step to pathogenesis

Morphological transitions are a major aspect of *H. capsulatum’s* capacity to cause disease as the inhaled conidia and mycelial fragments do not express several of the virulence factors observed in the yeast morphology. Temperature is by far the most exploited trigger to morphological changes. Medoff and colleagues investigated thermodimorphism during *H. capsulatum* growth using different human strains [75]. Remarkably, the Downs strain, a clinical isolate continuously passaged in laboratory [76] displayed a slower hypha-to-yeast transition *in vitro*, along with a slower ability to grow at 37°C when compared to more virulent strains. This temperature sensitivity was also correlated with a reduced virulence of the Downs strain in a murine model of histoplasmosis. Although morphological transitions are considered a key virulence factor for *H. capsulatum*, we cannot rule out that other environmental factors as well as differences in virulence between murine models and humans could be associated with these results [75].

In eukaryotic cells, cyclic adenosine monophosphate (cAMP) is a secondary messenger produced in response to extracellular stimuli and regulates a series of physiological process [77]. cAMP appears to be an important regulator of morphogenesis in *H. capsulatum*, as temperature shift promotes changes in cAMP intracellular levels and addition of exogenous cAMP and agents that raise the intracellular levels of cAMP arrest the fungus in its mycelial form even at 37 ºC [4,78]. Furthermore, cysteine and other sulfhydryl compounds are also important morphological regulators [5,79]. Once the temperature rises to 37°C, mycelial fragments and conidia decrease their metabolic activity [79]. This panorama only changes when mitochondrial respiration is reactivated by “shunt pathways” mediated by sulfhydryl compounds, such as cysteine. These pathways are operational at 25 ºC, but shut down during the early stages of morphologic transition, probably due to intracellular cysteine depletion [5,79]. Pharmacological blocking of sulfhydryl compounds can irreversibly impair the morphological transition. This treatment has no effect on fungal growth, suggesting that it specifically impacts *H. capsulatum* morphological transition. Altogether, these data confirm the importance of sulfhydryl compounds and can explain why mycelia are unable to cause disease in mice [80].

The impact of temperature and morphological changes in *H. capsulatum* have been investigated at a molecular level. Comparative transcriptome analysis between yeast and hyphal-phase using different *H. capsulatum* strains confirm that transcripts for genes encoding proteins previously identified as potential virulence factors, such as *CBP1 and YPS3*, are enriched in yeast-phase transcripts [7–9]. Cbp1 is a calcium binding protein, tailored to function in the phagolysosomal environment [81–84] and Yps3 is a yeast-phase specific protein that promotes dissemination from the initial pulmonary infection site [81,85]. Although *YPS3* was identified in yeast-phase transcripts of several strains [86], only some of them produce Yps3 [9,86]. This differential production seems to result from transcriptional regulation since the placing of an ectopic promoter in control of YPS3 was sufficient to restore Yps3 in strains initially unable to produce it [86]. The hybrid histidine kinase *DRK1* was directly associated with mycelia-to-yeast transition, sensing the host signals and being responsible for yeast phase growth and expression of other proteins such as Cbp1 [6].

Moreover, proteins from the *RYP* family, Ryp1, Ryp2, Ryp3, and Ryp4 have been identified as key regulators of the yeast-phase transcriptional program, regulating 96% of yeast-phase transcripts. These proteins seem to exert multiple functions, impacting genes not only capable of regulating cellular morphological changes, but also virulence, as well as each other’s expressions [8,87,88]. Recently, Rodriguez *et al*. performed the first genetic screen to identify genes required for hyphal growth at room temperature (RT) [89]. Remarkably, the disruption of *MSB2* resulted in a yeast-locked morphology at RT and also impacted the expression of *STU1* [89], a transcription factor from the APSES protein family, which is a largely conserved family among fungal species [90]. In addition, Ryp3 inhibits *MSB2* transcript expression at 37°C, whereas *MSB2* inhibits accumulation of Ryp transcripts at RT. The authors also theorize that Msb2 does not act directly on the expression or activity of Ryp, but rather functions indirectly, through other regulatory molecules such as Stu1 [89]. Together, these results indicate that Ryp and Msb2 are opposing pathways antagonizing each other in a temperature-dependent manner [89].

Understanding the genetic and molecular regulators and mechanisms involved in the mycelia-to-yeast transition, which seems to be the key for the successful infectious process, could lead to new prophylactic measures or to the development of therapeutic alternatives to tackle histoplasmosis at the onset of infection.

### Bypassing host barriers

In the environment, the mycelial form of *Histoplasma* can produce two types of conidia varying in size, named as macroconidia or microconidia [91]. The environmental significance of these different conidia are not yet fully understood, and although there is no data indicating that the infection occurs strictly from the inhalation of microconidia as opposed to macroconidia, some authors consider that, from a clinical perspective and based on the size of the particles, infection presumably occurs after the inhalation of microconidia and hyphal fragments that convert to budding yeast expressing virulence traits and causing disease [2,91].

After reaching the lungs, *H. capsulatum* face a group of pulmonary surfactant proteins (SP), including surfactant protein A (SP-A), SP-B, SP-C, and SP-D [11,92]. While SP-B and SP-C are responsible for maintaining low surface tension in the lung, SP-A and SP-D are termed collectins, soluble pattern-recognition receptors (PRRs) members of C-type lectins. Collectins are involved in a number of mechanisms related to defense against fungal, bacterial, and viral pathogens [92]. The role of surfactant proteins during histoplasmosis have been partially investigated. McCormack and colleagues studied the role of collectin SP-A and SP-D during the clearance of *H. capsulatum* [10]. Their results demonstrated that SP-A and SP-D displayed a direct fungicidal activity through a calcium-dependent mechanism of yeast permeabilization and, *in vivo*, the pulmonary clearance of *H. capsulatum* is reduced in SP-A null mice when compared to wild type [10]. Remarkably, the yeast internalization by macrophages impaired the antifungal activity of these collectins, suggesting that the intracellular environment protects the fungus against this innate barrier [10,11]. Recently, Carreto-Binaghi *et al*. measured the presence of collectins in human patients infected with *H. capsulatum* and healthy individuals [93]. Their analysis showed that *H. capsulatum* infection is associated with higher levels of SP-A while no significant differences are detected for SP-D [93]. Remarkably, in patients co-infected with HIV and *H. capsulatum*, the presence of SP-A was substantially reduced, suggesting a link between the SP-A depletion from the bronchoalveolar lavage and higher fungal burden. Such phenomena were not observed when patients were co-infected with HIV and *Pneumocystis jirovecii*.

### Evading the immune system

Studies have shown that during the first 7 days of infection, yeasts interact with epithelial cells, macrophages, dendritic cells, neutrophils, and natural killer cells [94–96]. The nature of these host–pathogen interactions is highly variable, but it is noteworthy that *H. capsulatum* yeasts are highly capable of surviving inside macrophages in contrast to other cell types, which turns out to be a key process for their dissemination. In this topic, we will discuss these interactions and their singularities, but mostly how *H. capsulatum* yeasts use macrophages as refuge to evade the immune system and establish disseminated infection.

#### Macrophages

Although resident macrophages are normally involved with an initial combat against fungal infections, during histoplasmosis this clash is critical. In fact, *H. capsulatum* yeasts are readily phagocytosed by resident alveolar macrophages in a process that does not require opsonization [13,97]. However, lung resident macrophages are not able to kill *H. capsulatum*. After internalized by macrophages, the yeasts prevent lysosomal acidification and phagolysosomal fusion, which allows *H. capsulatum* multiplication [15,18]. If successful, the yeast cells will continue to grow and divide within the macrophages, prolonging their intracellular life until they eventually induce host cell apoptosis and disseminate [98–100].

An early study performed by Bullock and Wright provided evidence that three different complement receptors expressed by human macrophages (CR3, CR4, and LFA-1) were involved in *H. capsulatum* binding to macrophages. The authors also noted that blocking the α subunit of any of these receptors did not impair recognition, theorizing that the recognition site was located on the common β subunit (CD18) of these receptors [101]. Further investigations by Newman *et al*. demonstrated that blocking the β subunit of these three receptors resulted in a 50% to 90% reduction of association between *H. capsulatum* yeasts and macrophages [74]. Additionally, Long *et al*. elegantly proved that the main receptor involved in *H. capsulatum* internalization by human macrophages is CR3 (CD11b/CD18). In their studies, CR3 expression in Chinese hamster ovary cells lacking the CD18-family integrins led to a substantial increase in *H. capsulatum* binding [13]. They also showed that the recognition of *H. capsulatum* by CR3 is mediated by the 60 kDa Heat Shock protein (Hsp60) [13].

Recently, our group demonstrated the relationship between the organization of lipid microdomains (LD) and the process of *H. capsulatum* yeast internalization by macrophages [102]. LDs are dynamic and highly organized regions of the plasma membrane that contain or recruit pattern recognition receptors during pathogen–host interaction [103]. LD favor recruitment and clustering of receptors and co-receptors impacting ligand recognition and signaling transmission between pathogens and host cells [104]. We demonstrated that disruption of LD through cholesterol depletion using with methyl-*β*-cyclodextrin (*m*-β-CD) slowed the rate of yeast-macrophage stable adhesion and reduced internalization of *H. capsulatum* yeasts by macrophages. These results suggested that *H. capsulatum* internalization is a process highly dependent on the assembly of these platforms. Our results also indicated that the GM1 glycosphingolipid is involved in this process as an accessory molecule [102], as pharmacological inhibition of complex gangliosides synthesis or the use of peritoneal macrophages from mice lacking them (*B4galnt1*^−/−^), such as GM1, had its ability to recognize H. *capsulatum* yeasts impaired [102]. Additionally, we also demonstrated that both GM1 and CR3 are translocated to the *H. capsulatum* binding sites at the macrophage cell surface. Finally, CD18 mobilization to lipid microdomains was reduced in macrophages from *B4galnt1*^−/−^. It appears that LD assembly is relevant for a correct and stable recognition of *H. capsulatum* by macrophages [102,103]. The absence of LD components apparently delays the integrin mobility, modifying the adhesion kinetic and consequently, the dynamic of fungal internalization, a process in which, the involvement of lipid rafts and associated protein-lipid diffusion has been implied [105].

Despite its ability to bind CR3, Hsp60 is a major *H. capsulatum* virulence factor and has a potent immunogenic activity [12,14–16]. Gomez *et al*. showed that vaccination with recombinant Hsp60 was able to protect mice challenged with a lethal inoculum of *H. capsulatum* [12]. Subsequently, our group demonstrated that monoclonal antibodies (MAbs) to Hsp60 displayed a protective effect *in vitro* and in a murine model of histoplasmosis [14]. The efficacy of these MAbs were subclass-dependent, according to the affinity of each mAb with its Fcγ receptor [16]. IgG1 and IgG2a MAbs could interact strongly with Fcγ receptor I and Fcγ receptor III (FcγRIII) facilitating phagocytosis and enhancing fungal killing, thus demonstrating protective effect, while other MAbs, that interacted strongly with inhibitory Fcγ receptors, resulted in low or no microbicidal responses by macrophages [16]. In addition, Hsp60 is a key protein during the orchestration of a heat shock response. In response to temperature stress Hsp60 levels significantly increased in the cytoplasm and cell wall subcellular fractions, interacting with both essential and non-essential proteins as a key regulator of diverse cellular processes, including amino acid, protein, lipid, and carbohydrate metabolism, cell signaling, replication, and expression of virulence associated proteins [15].

**Figure 2.**
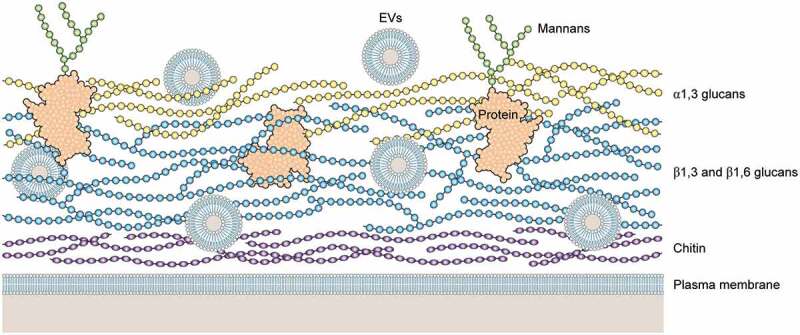
Schematic illustration of a hypothetical model of H. capsulatum cell wall. Cell wall of H. capsulatum consists primarily of polysaccharides, including chitin (dark blue), β1,3 and β1,6 glucans (light blue), a1,3 glucans (yellow), proteins (black coil) and mannoproteins (black coil with mannans in green). Extracellular vesicles (EVs) are shown as bilayered compartments. For didactic purposes, minor components such as lipids are not shown.

Recognition of Hsp60 by CR3 is not the only strategy used by macrophages to internalize or respond to *H. capsulatum*. Macrophages have a combination of cell wall receptors in place, with the ability to recognize pathogen-associated molecular patterns (PAMPs). For instance, the C-type lectin Dectin-1, an important receptor in the recognition and immune response to fungal pathogens [106–108], recognizes β-1,3-glucan, a major polysaccharide in the cell wall of fungal pathogens, including *H.* *capsulatum* (Figure 2) [42]. Although not directly involved in phagocytosis, Dectin-1 engagement to *H. capsulatum* mediates cytokine production in a mechanism dependent on CR3 association [109]. Huang and colleagues demonstrated that co-localization of Dectin-1 and CR3 into GM1 microdomains at the binding interface stimulates the production of TNF and IL-6 by macrophages [110]. Some strains of *H. capsulatum*, express an outer layer containing α-1,3-glucan [19,42]. This polysaccharide impairs the recognition of β-1,3-glucan by Dectin-1 [17,19,111]. Depletion of the α-1,3-glucan synthase AGS1 through RNA interference reduces the α-1,3-glucan layer and decreases the fungal virulence [112], reinforcing the relevance of dectin-1 recognition during the innate immune response against *H. capsulatum*.

The ability to produce α-1,3-glucan is also a particular aspect to determine *H. capsulatum* chemotypes I and II. The chemotype II strains have the α-1,3-glucan outer layer in their cell wall, right above the β-1,3-glucan layer, whereas chemotype I strains have only the β-1,3-glucan layer [19,42]. Interestingly, the strains that lack α-glucan show no difference in their virulence, evidencing that both chemotypes have mechanisms to evade detection by Dectin-1 [19]. Chemotype II strains kill macrophages faster resulting in a shorter course of infection, whereas chemotype I strains, although slower at killing macrophages, reach higher fungal burdens overall, revealing different virulence strategies [113]. A series of studies performed by Garfoot *et al*. provided insights regarding the potential mechanisms used by these strains lacking α-glucan [20,111]. The protein Eng1, a member of the glycosyl hydrolase 81 family, secreted exclusively by the yeast-phase *H. capsulatum*, seems to be a key factor in evasion from Dectin-1 recognition. *H. capsulatum* yeasts with reduced levels of *ENG1* exhibit attenuated virulence *in vivo*, *w*hereas partial depletion of *ENG1* is associated with a higher exposure of β-1,3-glucans and a consequent enhancement of Dectin-1 binding, triggering typical pro-inflammatory cytokine response (TNF- α and IL-6) [111]. It is important to point out, that similar results were observed in α-glucan producing strains, suggesting that Eng1 cooperates with α-glucan, removing any β-glucan that is not masked by it [111]. Garfoot *et al*. also determined the enzymatic specificity of Eng1 and E×g8, confirming that both enzymes hydrolyze exposed β-1,3-glucans in the cell wall with endoglucanase and exoglucanase activity, respectively [20]. In addition, their results showed that, besides E×g8 having a different specificity for β-1,3-glucans, this enzyme was not effective in reducing host detection by Dectin-1, as it was largely dispensable for virulence *in vivo* [20]. Taken together, these results demonstrated that Eng1 activity is the predominant factor responsible for removal of exposed cell wall β-1,3-glucans, minimizing *H. capsulatum* recognition by Dectin-1, as E×g8 exoglucanase is dispensable, suggesting that the exposed β-1,3-glucans chains lack exposed termini for its activity [20].

Together, β-1,3-glucanases and the α-1,3-glucan layer avoid recognition by Dectin-1, without compromising Hsp60-CR3 engagement. However, this considerably impacts the internalization outcome, leading to yeast internalization without triggering pro-inflammatory cascades. Once inside macrophages, *H. capsulatum* is able to survive and grow, exploiting a series of mechanisms, including the ability to inactivate oxygen and nitrogen reactive species, and preventing phagosome acidification and lysosomal fusion [18].

The first intracellular response to *H. capsulatum* is an oxidative burst caused by activation of the membrane-associated NADPH-oxidase complex, responsible for reducing oxygen to superoxide at the phagosomal membrane [114]. Superoxide and other molecules derived from it, which include peroxide and hydroxyl radicals, are collectively called reactive oxygen species (ROS), and since they are highly toxic and capable of directly damaging the invading microbe, pathogens must avoid or neutralize ROS in order to survive [19,22,115]. As expected, being an effective intracellular pathogen, *H. capsulatum* is well known for enduring high concentrations of ROS [116–118]. Holbrook *et al*. identified three proteins potentially involved with the *H. capsulatum* anti-oxidative response [21]. Superoxide dismutase 3 (Sod3), in particular, is upregulated (approximately 100-fold) in the yeast form, suggesting that morphological changes are accompanied by an increased ability to survive in oxidative environments [21,22]. Using a strain lacking the *SOD3* gene, Youseff *et al*. investigated the contribution of Sod3 to *H. capsulatum* pathogenesis [22]. Their results confirmed that Sod3 is required to promote resistance against superoxide and they also highlighted that even in a resting state, infected macrophages continue to produce superoxide, in contrast with early studies suggesting that *H. capsulatum* does not trigger oxidative burst in all infected macrophages [22,119]. Regulation of ROS production starts when PRRs recognize PAMPs at the fungal cell surface. Nevertheless, activation of macrophages by cytokines primes these cells to produce ROS, and the *sod3Δ* mutant strain of *H. capsulatum* triggered relatively low levels of it, which may result from the decreased phagocyte recognition mediated by the masking of cell wall β-glucans as previously described, this observation is supported by the fact that other pathogenic yeasts, *eg. C. albicans*, which lack a mechanism to evade recognition via Dectin-1, have a significant increase in ROS production caused by the signaling cascade mediated by this receptor [22,28,120–122]. Furthermore, Holbrook *et al*. investigated the role of catalases CatB and CatP in oxidative stress defense, with extracellular and intracellular activities, respectively. Investigating mutants lacking those enzymes, the authors observed that both CatB and CatP seem to protect *Histoplasma capsulatum* from hydrogen peroxide challenge *in vitro* and from ROS produced by human neutrophils [23]. It was also noted that optimal protection required both catalases, as the double mutant yeasts had a lower survival rate than single-catalase mutants, and that these catalases display some degree of redundancy, as loss of both CatB and CatP attenuated virulence *in vivo*, but loss of only CatB did not significantly alter the course of lung infection and extrapulmonary dissemination [23].

Superoxide combines with nitric oxide (NO), generating peroxinitrite (ONOO–) [114], a reactive nitrogen species (RNS) that causes DNA and membrane damage, disrupts cell replication and respiration, and inactivates cellular enzymes [123]. NO is required to control *H. capsulatum* replication within the intracellular space [114,123]. This effect has been mostly described as fungistatic [74,124,125]. To give further insight into the mechanisms by which *H. capsulatum* resists to RNS, Nittler *et al*. identified *NOR1* as a gene up regulated after RNS exposure. This gene encodes a homologue of a nitric oxide reductase produced by *F. oxysporum* (P450nor) [123]. P450nor is an enzyme related to denitrification, a process used by soil microbes to generate energy under anaerobic conditions. During this metabolic process, P450nor reduces NO to the non-toxic nitrous oxide [126–128]. Interestingly, *H. capsulatum NOR1* is induced in both the mycelial and yeast phases of *H. capsulatum*, but *NOR1* is specifically upregulated in the yeast phase in the presence of NO [123]. This probably occurs because the mycelial form of *H. capsulatum* grows in soils enriched in nitrogen and may perform Nor1-dependent denitrification. Based on these results, the authors hypothesized that *H. capsulatum* yeasts have co-opted an enzyme normally used in its saprophytic phase to overcome hosts defense mechanisms [123].

In addition to evading reactive oxygen and nitrogen species, *H. capsulatum* yeasts are able to regulate phagosome acidification and prevent lysosomal fusion as part of their strategy to survive in the intracellular space [129]. When *H. capsulatum* is internalized by peritoneal macrophages or by the macrophage-like cell-line J774.2, phagolysosomal fusion can occur, but the process is reduced at higher infection ratios [130]. This process was further explored by Eissenberg *et al*. using the macrophage-like cell-line P388D1.D2. According to these authors, phagolysosomes from infected P338D1.D2 macrophages displayed a higher pH, influencing the organelle antifungal activity [131]. The authors also noted that the fungal cell must be alive in order to control the phagolysosomal pH [131]. In contrast, in RAW264.7 macrophages, a murine derived cell line, *H. capsulatum* controls the pH using a distinct mechanism. In these cells, *H. capsulatum* inhibited normal phagolysosomal fusion and dampened the accumulation of V-ATPase [129]. This phenomenon only occurs when the pH reaches a value where the yeast can access iron from transferrin (pH 6.5) and this acidification seems to be controlled by *H. capsulatum* [129]. Therefore, RAW267.4 appears to behave similarly to human macrophages, where phagolysosomal fusion in inhibited [129]. It is possible that phenotypic changes in each macrophage could impact the outcome. In fact, Lin et al. compared the cell surface receptors expression in thioglycollate-elicited peritoneal macrophages, residential peritoneal macrophages, bone marrow-derived macrophages and RAW267.4 macrophages. Although CR3 was similarly expressed by all macrophages investigated, Dectin-1, TLR2 and, specially, the mannose receptor displayed a significant variability [109]. Although the major receptor for H. capsulatum yeasts is CD18, it is possible that other co-receptors are involved in regulating and impacting the intracellular response, and it is also important to highlight that the molecular mechanisms by which *H. capsulatum* controls the phagosome environment are not clear. Isaac *et al*. showed that macrophage internalization was followed by phagolysosomal acidification with *H. capsulatum* lacking *HCL1*, which encodes the enzyme HMG CoA lyase. This highlights the importance of the regulation of phagosomal acidification by *H. capsulatum*, as this mutant strain had impaired intracellular growth, lacked the ability to lyse the phagocytes, and, consequently, was also less virulent in the murine model of infection [132]. Nevertheless, further investigation in this area is required to elucidate the mechanisms by which *H. capsulatum* is able to modulate acidification and especially prevent lysosomal fusion.

*H. capsulatum* yeasts use macrophages to disseminate to other tissues. Further, yeast cells may induce apoptosis or somehow leave the infected macrophage and move to a subsequent phagocyte. Deepe and Buesing demonstrated that upon infection, an increased expression of TNF-α and subsequent activation of caspases 3 and 1 are involved in the process, with both contributing to the process of cell death, but only caspase 1 involved in apoptosis [99]. The authors also showed an increase in the IL-10 production, and this cytokine inhibits apoptosis providing a niche for yeast to keep growing [99]. The chronology of TNF-α and IL-10 release differed *in vitro*, with the latter being detected only 4 hours after the release of TNF-α, thus leading the authors to hypothesize that the late release of IL-10 is likely to prevent apoptosis of newly infected cells [99]. This anti-apoptotic effect was observed in earlier studies. Medeiros *et al*., demonstrated that *H. capsulatum* was capable of inducing an anti-apoptotic state on different leukocytes, suggesting that the yeasts are highly capable of regulating the apoptotic and anti-apoptotic effects as suitable for the infection process [100]. Although this is an area that requires further exploration, Pitangui *et al*. reported an intracellular arrangement of *H. capsulatum* yeasts consisting of aggregates surrounding the macrophage nucleus, and they hypothesized that this arrangement was able to cause damage to nuclear DNA and induce apoptosis in alveolar macrophages. Thus, the observed nuclear damage, occurring while yeast cells were not located inside the nucleus, could be part of *histoplasma’s* infection and intracellular survival strategy [98].

The correlation between molecules produced by *Histoplasma* and host cell death has been investigated by Isaac *et al.*, the authors found three mutants, lacking *CBP1* expression, while searching for mutants that would thrive in the intracellular space, but without lysing the host cells [24]. The calcium-binding protein (Cbp1) is a yeast-phase protein involved with the cell growth under calcium limited environments and early studies demonstrated that Cbp1 also plays a role in cell proliferation, as *CBP1* mutants are unable to grow in a calcium deprived environment or proliferate in a murine model of pulmonary infection [83,133]. Additionally, Isaac *et al*. demonstrated that *Histoplasma* actively triggers macrophage death when producing *CBP1*, as its mutants continue to grow in the intracellular space without causing macrophage death in a passive manner. The authors also showed that *CBP1* is required for induction of integrated stress response, involved in the activation of caspases 3 and 7 [24]. In a posterior study, the authors investigated the cell death induced by Cbp1 and the latter finding was supported by data demonstrating that the induction of the transcription factor *CHOP* and the pseudokinase *Tribbles 3* (*TRIB3*) by Cbp1 is essential for triggering apoptosis [25]. Additionally, macrophages lacking *CHOP* or *TRIB3* are resistant to cell lysis and *CHOP* knockout mice are not only resistant to *H. capsulatum* infection, but display a diminished fungal burden compared to their wild-type counterparts [25]. More recently, the same research group also demonstrated that *H. capsulatum* Cpb1 accesses the macrophage cytosol during infection, suggesting the cytosol of the host cell as the site of action where Cpb1 would actively induce an integrated stress response [26]. The authors also demonstrated that Cbp1 binds to another important *H. capsulatum* protein, Yps3, and that the deletion of Yps3 resulted in diminished host-cell lysis, suggesting that this protein could potentiate the function of Cbp1 [26].

#### Dendritic cells

Dendritic cells (DCs) are professional antigen-presenting cells, capable of processing and presenting antigenic peptides to T cells [134], acting as a bridge between the innate and adaptive immune response [134,135]. In the lung alveoli, *H. capsulatum* yeasts are recognized and readily phagocytosed by DC, and (differently from macrophages) internalization by DC leads to phagosome/lysosome fusion and the destruction of the yeasts within hours [136,137].

In a series of studies, Gildea *et al*. demonstrated that *H. capsulatum* recognition by DC is mediated by the fibronectin receptor, an integrin called very late antigen-5 (VLA-5) at DC surface rather than the CD18 receptors, described for macrophages [136]. It was a particularly interesting finding since both DC and macrophage express CD18 and VLA-5 in quantities that are roughly equivalent [136]. The reason why *H. capsulatum* uses one receptor preferentially is still unknown, but these authors hypothesized that this may be the key to the antagonistic destinies in these phagocytes, as macrophages are far more permissive to *H. capsulatum* intracellular growth. The authors also hypothesize that phagocytosis via VLA-5 may trigger an intracellular signaling cascade that allows DC to counter the ability of *Histoplasma* yeasts to inhibit or reduce phagolysosomal fusion. In fact, attempting to understand the mechanism by which DC kills *H. capsulatum*, Gildea *et al*. found that in DC phagolysosomal fusion occurs normally [137], in contrast to the minimal amount of phagolysosomal fusion that occurs in human macrophages [138]. Additionally, DC action can be both fungicidal and fungistatic with oxidative burst playing a major role but, as opposed to macrophages, mainly via hydrolases rather than nitric oxide and ROS as previously described in this review [137]. Later, Gomez *et al*. identified the ligand for VLA-5 on the surface of *H. capsulatum* yeast cells as cyclophilin A [27].

Other receptors have been associated to cellular responses when *H. capsulatum* interacts with DC. For instance, Dectin-1 and Dectin-2 are involved with NLRP3 inflammasome activation through a collaborative relationship. Dectin-2, which recognizes mannosylated ligands, appears to be the primary receptor and Dectin-1 the secondary one, probably because of the β-glucan masking mechanisms previously described. Notably, IL-1*β* production is abrogated only when both receptors are deleted [139]. In addition, TLR7 and TLR9 are key receptors, for which the *Histoplasma* ligand is unknown, to induce IFN-I production when DC are incubated with *H. capsulatum* [140]. During murine histoplasmosis, these receptors are required for an appropriate adaptive immune response [140].

#### Neutrophils

Neutrophils usually represent the first-line cells recruited against invasive fungal infections [116,117]. Their role during histoplasmosis is still controversial. Deepe *et al*. investigated the population of phagocytic cells involved with *H. capsulatum* phagocytosis during the first week of infection, where only the innate immune response is active [95], and demonstrated yeasts were within macrophages, polymorphonuclear leukocytes (PMN) and DCs. The percentage of yeasts in macrophages remained similar over time, while in PMN they were higher and in DCs they reduced over the course of the experiment. Thus, neutrophils could contribute to histoplasmosis control. Using the same *H. capsulatum* strain (G217) and mouse background (C57BL/6), Baughman and colleagues demonstrated that PMN along with lymphocytes and macrophages were indeed the major cells characterized one week after intranasal infection in a murine model of histoplasmosis [141]. However, this set of cells recruited to the site of infection were not able to control fungal growth, since a significant number of viable yeasts were found. The numbers of PMN decreased at day fourteen and lymphocytes and macrophage became more prominent. Granulomas and chronic inflammation were observed along with a reduced of viable yeasts. These data initially suggested that neutrophils are not crucial to control histoplasmosis [141]. On the other hand, a number of *in vitro* studies investigated the battle between *H. capsulatum* and neutrophils *in vitro*, have established important fungicidal and fungistatic activities in neutrophils [142,143]. Newman *et al.* demonstrated that the major receptors at the neutrophil surface involved with *H. capsulatum* internalization are the complement receptors 1 and 3 (CR1 and CR3) and FcRIII [142]. Using subcellular fractions of neutrophils, these authors confirmed that the compounds involved with the antifungal effect were localized in the azurophilic granules [142]. The azurophilic granule contents consist mainly of proteins from two distinct families (defensins and serprocidins) and the bactericidal-permeability-increasing protein (BPI) [142,143]. In subsequent work, Newman *et al.* demonstrated effectively that defensins released by neutrophils inhibit yeasts growth *in vitro* in a dose-dependent fashion [143]. Cathepsin G and BPI also inhibit growth of *H. capsulatum* displaying an additive effect between each-other and defensins [143]. It was also hypothesized that, in immunocompetent individuals, the initial response from neutrophils may be sufficient to clear the fungal burden without the need for activation of the adaptive immune response, which is supported by the fact that the majority of individuals from areas where histoplasmosis is endemic had negative skin test reactions when exposed to histoplasmin [142].

Using confocal fluorescence and scanning electron microscopy techniques, Thompson-Souza *et al*. elegantly demonstrated the release of neutrophil extracellular traps (NETs) in response to *H. capsulatum in vitro* [144]. NETs are structures of chromatin filaments coated with granular and cytosolic proteins, histones, and proteases, these structures help neutrophils immobilize a series of pathogens, including fungi, creating a microenvironment that is detrimental to the fungal infection [144–146]. The process was associated with the CD18-dependent activation of Syk and Src kinase pathways. NET release displayed a fungicidal activity against *H. capsulatum* yeasts [144]. Taken together, the mechanisms described above could explain how neutrophils create an adverse microenvironment when *H. capsulatum* yeasts are not in the relatively safe intracellular environment, trapping and killing them.

#### Natural killer

Little is known about the involvement of NK cells in histoplasmosis. Like dendritic cells, natural killer cells have a cytotoxic effect and act as a bridge between innate and adaptive immunity. When activated by IL-12 produced by macrophages and dendritic cells, NK cells produce IFN-γ. This, in addition to the subsequent activation of CD8 and CD4 T cells, characterizes part of the immune response taking place in order to control infection or eventually form granulomas [147,148]. Early studies implied the involvement of NK cells in the immunological response to *Histoplasma* infection, as Cain and Deepe monitored the influx of different cells and their respective cytokine production in the first days of infection, showing a peak of NK cells in the seventh day of infection [94].

Cohen *et al*. proposed a general mechanism for NK cells function during systemic fungal infection where yeast cell wall components, such as β-glucan, stimulate APCs to produce IL-12, triggering NK cell activation [149]. This is supported by the findings demonstrating that DC associated with *H. capsulatum* yeasts were unable to produce IL-12 resulting in a decreased NK response [149]. Taken together, these data provide evidence that NK cells are involved in the control of histoplasmosis and that recognition of cell wall β-glucans plays a direct role in NK cell response.

#### Adaptive immune response

Under specific conditions, including high fungal loads, or when the development of a consistent innate response is impaired, fungal clearance is achieved through effective activation of adaptive immune response. The interaction between antigen presenting cells and T lymphocytes will, eventually, generate a protective Th1 immune response, with the production of IL-12, IFN-γ and TNF-α [150]. During histoplasmosis, the formation of granuloma is frequent [151–154], but it does not necessarily result in the eradication of the infection. In fact, the granuloma will serve as interface between host immune cells and *H. capsulatum* [151].

Heninger *et al*. were the first to closely analyze this interface and provided evidence that the infection-associated lesions contain both CD4+ and CD8+ T cells, and that they are the primary source of IFN-γ and IL-17 associated with these lesions [151]. IFN-γ activates macrophages, inducing oxidative burst as described previously, and, although *H. capsulatum* is highly capable of counter it, series of studies have shown the importance of this cytokine for disease management, as depletion and gene disruption resulted in an increase in virulence in the murine model [155].

Yeasts of *H. capsulatum* may remain viable within the granuloma, and can be reactivated as a result of impaired immunity [154]. Functional T and B cells are essential to prevent reactivation in murine model, as depletion of CD4+ and CD8+ at the same time, as well as depletion of T cells in B cell-deficient mice resulted in infection persistence [154]. The authors also demonstrated that a fine regulation of cytokine production is involved with reactivation of histoplasmosis, as treatment with anti-IFN-γ, anti-TNF-α, or both did not induce reactivation, whereas endogenous IL-10 exacerbated the process [154].

### Nutrient acquisition

Being a pathogen highly adapted to live intracellularly, *H. capsulatum* yeasts effectively acquire essential nutrients inside phagocytic cells, especially in macrophages, where the pathogen has multiple mechanisms that allow its survival [156].

Glucose is the main carbon source used in culture media as it reflects some of the environments found in the host. Other niches, such as the intracellular environment, especially the phagosome, are characterized by alternative carbon sources [156]. In fact, Shen *et al*. carefully demonstrated that *Histoplasma* yeasts do not catabolize hexoses in the intracellular environment. The authors identified that genes encoding enzymes such as phosphofructokinase (Pfk-1) and hexose/glucose kinases (*HXK1* and *GLK1*) were down-regulated during macrophage infection by *H. capsulatum* yeasts. Evidence was obtained when depletion of these enzymes impaired growth in glucose-rich media while not affecting *H. capsulatum* growth in macrophages *in vitro*, nor impacting infection *in vivo* [157]. Furthermore, they also showed up-regulation of *PCK1* gene by *Histoplasma* yeasts within macrophages, which encodes a phosphoenolpyruvate carboxykinase and is related to the first step of gluconeogenesis. Elimination of Pck1 activity prevented yeast growth in macrophages *in vitro* and severely attenuates the disease in murine model. Taken together, these findings indicate that *H. capsulatum* relies on metabolizing gluconeogenic carbon sources within macrophages [157].

*H.capsulatum* yeasts can also use amino acids as a non-carbohydrate carbon source. The phagosome/phagolysosome is a degradative organelle that contains several proteases capable of generating amino acids and short peptides to the pathogen within [156]. It is not known which amino acids are available within the phagosome and which are catabolized by the intracellular yeasts, but Shen *et al*. also demonstrated that the gene expression between yeasts growing on amino acids *in vitro* were highly similar to that of yeasts grown within the macrophage. Additionally, yeasts metabolize amino acids after digestion by proteinases, such as Cathepsin D, but cannot utilize intact proteins such as bovine serum albumin and hemoglobin as substrate for carbon source [157].

Although in relatively smaller amounts, vitamins are also needed for fungal growth. Garfoot *et al*. demonstrated that *Histoplasma capsulatum* yeasts can synthesize all essential vitamins with the exception of thiamine, they also generated riboflavin, pantothenate, and biotin auxotrophs to probe their availability in the intracellular milieu. Their results demonstrated that disruption of riboflavin and pantothenate biosynthesis prevented growth and proliferation of yeasts, severely attenuating virulence in the murine model of histoplasmosis, evidencing that the phagosome environment limits the yeast access to these vitamins [158].

Trace metals, such as iron, copper, and zinc present a challenge to an intracellular pathogen such as *H. capsulatum* because host cells can limit their availability and yeasts cannot synthesize them [159]. While iron, copper, and zinc are sufficiently abundant in resting macrophages, activation of these host cells causes a restriction of these elements as a form of nutritional immunity, creating a scenario where *Histoplasma* yeasts need to use various mechanisms to battle these restrictions and survive in the intracellular space [159].

*H.capsulatum* secretes siderophores for scavenging iron [160,161]. Free iron levels are usually kept low in the human body due to binding by several iron chelators, such as transferrin, and this is a mechanism by which extracellular microbial proliferation is controlled, as the necessity of iron as a nutritional requirement has been shown for many pathogens [162–164]. However, this same mechanism is what probably facilitates Iron acquisition by intracellular pathogens, such as *H. capsulatum* [159,162], as trafficking of holo-transferrin into phagosomal compartments causes the release of its iron content due to the acidic environment [162]. In fact, a series of studies demonstrated that *Histoplasma* iron acquisition from the host is dependent on phagolysosomal pH [129,165], with *H. capsulatum* yeasts acquiring iron directly from transferrin, through acidification of the phagolysosome, once again highlighting the importance of pH modulation by *Histoplasma* yeasts to their survival [165]. Additional studies also noted that the impairment of siderophores biosynthesis diminishes *H. capsulatum* growth within macrophages and results in attenuated disease in the murine model of infection [161]. However, it was further noted that siderophores were not required for proliferation until 2 weeks of infection, which could indicate that during the early stages of infection, iron is abundant within the phagosome and only becomes a limiting factor after activation of host cells [156,161].

Similarly, copper is another trace metal that has different levels of availability depending on the macrophage activation status. Shen *et al*. found that IFN-γ activation of phagocytes causes the restriction of phagosomal copper, and that the copper transporter Ctr3 is required for intracellular proliferation, as Ctr3-defective yeasts were partially attenuated during infection, especially after the onset of adaptive immunity [29]. Corroborating these observations, the expression of *CTR3* is increased in activated macrophages, a low copper concentration environment but not in unactivated macrophages [29]. This data suggest that the activation of macrophages induces copper limitation as a defense mechanism from intracellular infection, which *H. capsulatum* yeasts counter with the production of Ctr3 [29].

Zinc is also essential to the development of histoplasmosis and, similarly to iron, its levels within the phagosome can change depending on the activation status of macrophages [156]. Recently, studies have shown that zinc becomes limited as macrophages are activated by the cytokine GM-CSF, and that *H. capsulatum* yeasts are capable of producing a zinc transporter enabling it to acquire zinc from the environment and to bypass this limitation [166,167]. Rossi *et al*. provided further evidence of the importance of zinc on the establishment of histoplasmosis. While treating macrophages with the glucose analog 2-deoxy-D-glucose (2-DG), the authors unexpectedly discovered that 2-DG diminished import of zinc into macrophages, decreasing the quantity of both cytosolic and phagosomal zinc, resulting in yeast cell death as a result of zinc starvation [168]. The authors highlighted that further studies should consider 2-DG as a possible adjunctive agent in histoplasmosis therapy [168].

Taken together, the studies reviewed above emphasize that the different strategies that *H. capsulatum* employs to acquire nutrients are absolutely necessary for the fungus to thrive in the intracellular environment of macrophages, being an integral part of pathogenesis, as without those, the establishment of the niche within these cells would fail, preventing the development of histoplasmosis.

## Extracellular vesicles: A novel virulence factor

Fungal extracellular vesicles (EVs) are bilayered compartments that are virtually released by all living cell types, including fungal organisms [169–174]. Fungal EVs composition includes lipids, polysaccharides, glycans, nucleic acids, proteins, pigments, and a variety of metabolites [175–177]. Based on their composition, it has been theorized that fungal EVs could be involved in multiple processes impacting directly and indirectly the course of infection. In fact, over the last decade, several functions for fungal EVs have been explored with contrasting results varying from one pathogen to another [176–180] Although it was the second fungal species where EVs were characterized, little is known about the biological role of EVs from *H. capsulatum* in histoplasmosis [169]. The EVs released by *H. capsulatum* carry a variety of molecules including, in a smaller fraction, a combination of virulence factors that suggest their involvement in disease developments such as Hsp60 and the enzymes catalases and superoxide dismutase [169,181]. Indeed, proteins carried by *H. capsulatum* EVs proved to be immunoreactive to histoplasmosis patients’ serum, demonstrating the presence of several virulence factors and the immunobiological potential of EVs produced by this species [169]. Remarkably, the first studies showing that environmental conditions and products of the immune response can modify the composition of EVs were published using *H. capsulatum* as prototype. By treating *H. capsulatum* yeasts with anti-Hsp60 mAbs, Baltazar *et al*. demonstrated significant changes in the EV cargo [181]. These results could help to explain the mechanisms involved in the protection conferred by mAbs in the murine model of histoplasmosis [14]. EVs produced by antibody-treated *H. capsulatum* have a distinct immunomodulatory effect on macrophages than EVs produced by non-treated *H. capsulatum*. EVs released by yeasts treated or not with mAb were incubated with macrophages resulting in a dampening of phagocytic capacity of 60% and 35%, respectively, thus providing some insight into *H. capsulatum* EVs pathophysiological role [182]. Furthermore, Cleare et al. demonstrated that the *H. capsulatum* EV cargo and release are highly influenced by different nutritional milieus. The authors highlight that these findings are an important step in providing indication that different host and environmental conditions are capable of modulating EV loading and release, and more specifically, that these changes could lead to different disease outcomes [183].

## Final considerations

Histoplasmosis is a disease that occurs worldwide, with presentations varying from mild symptoms to disseminated disease in its most severe state, the latter usually taking place in immunocompromised individuals. In its infective form, *H. capsulatum* is a yeast highly adapted to the intracellular environment and has numerous mechanisms by which it evades and resists the host’s defenses. Understanding and further investigating these mechanisms are essential in the search for new preventative and therapeutic treatment approaches as well as research strategies. The mechanisms discussed above also highlight the importance of building a comprehensive *H. capsulatum* mutant library, which would be pivotal to the efforts of characterizing virulence factors and novel antifungal targets.
